# Optimizing Sequential Systemic Therapies for Advanced Hepatocellular Carcinoma: A Decision Analysis

**DOI:** 10.3390/cancers12082132

**Published:** 2020-07-31

**Authors:** Giuseppe Cabibbo, Ciro Celsa, Marco Enea, Salvatore Battaglia, Giacomo Emanuele Maria Rizzo, Stefania Grimaudo, Domenica Matranga, Massimo Attanasio, Paolo Bruzzi, Antonio Craxì, Calogero Cammà

**Affiliations:** 1 Department of Health Promotion Sciences Maternal and Infant Care, Section of Gastroenterology & Hepatology, Internal Medicine and Medical Specialties, PROMISE, University of Palermo, 90127 Palermo, Italy; giuseppe.cabibbo78@gmail.com (G.C.); celsaciro@gmail.com (C.C.); g.rizzo.gr@gmail.com (G.E.M.R.); stefania.grimaudo@unipa.it (S.G.); antonio.craxi@unipa.it (A.C.); 2 Department of Surgical, Oncological and Oral Sciences (Di.Chir.On.S.), University of Palermo, 90127 Palermo, Italy; 3 Department of Health Promotion Sciences Maternal and Infant Care, Internal Medicine and Medical Specialties, PROMISE, University of Palermo, 90127 Palermo, Italy; marco.enea@unipa.it (M.E.); domenica.matranga@unipa.it (D.M.); 4 Dipartimento di Scienze Economiche, Aziendali e Statistiche, University of Palermo, 90133 Palermo, Italy; salvatore.battaglia91@gmail.com (S.B.); massimo.attanasio@unipa.it (M.A.); 5 U.O. Epidemiologia Clinica, IRCCS Ospedale Policlinico San Martino, 16132 Genova, Italy; bruzzipaolo49@gmail.com

**Keywords:** hepatocellular carcinoma, systemic therapy, sequential therapy, tumor progression, survival

## Abstract

*Background:* An optimal sequential systemic therapy for advanced hepatocellular carcinoma (HCC) has not been discovered. We developed a decision model based on available clinical trials to identify an optimal risk/benefit strategy for sequences of novel systemic agents. *Methods:* A Markov model was built to simulate overall survival (OS) among patients with advanced HCC. Three first-line (single-agent Sorafenib or Lenvatinib, and combination of Atezolizumab plus Bevacizumab) followed by five second-line treatments (Regorafenib, Cabozantinib, Ramucirumab, Nivolumab, Pembrolizumab) were compared in fifteen sequential strategies. The likelihood of transition between states (initial treatment, cancer progression, death) was derived from clinical trials. Life-year gained (LYG) was the main outcome. Rates of severe adverse events (SAEs) (≥grade 3) were calculated. The innovative measure, called incremental safety-effectiveness ratio (ISER), of the two best sequential treatments was calculated as the difference in probability of SAEs divided by LYG. *Results:* Lenvatinib followed by Nivolumab (median OS, 27 months) was the most effective sequence, producing a LYG of 0.75, while Atezolizumab plus Bevacizumab followed by Nivolumab was the safest sequence (SAEs 40%). Accordingly, the net health benefit assessed by ISER favored Lenvatinib followed by Nivolumab, compared to Atezolizumab plus Bevacizumab, followed by Nivolumab in 52% of cases. *Conclusion*: Further sequential clinical trials or large-scale real-world studies may prove useful to evaluate the net health benefit of the best sequential treatment for advanced HCC.

## 1. Introduction

Hepatocellular carcinoma (HCC) is characterized by clinical and biological heterogeneity [1,2]. In addition, most cases of HCC have a dismal prognosis that is strictly linked to the stage of cirrhosis [3]. Unfortunately, more than half of all cases of HCC are diagnosed at a stage with no potentially curative treatment options. Since 2008, the oral multikinase inhibitor (MKI), Sorafenib, has been recommended as the standard first-line systemic therapy for patients with Barcelona Clinic Liver Cancer (BCLC) C (advanced) HCC, and also for patients with BCLC B (intermediate) HCC, which have been deemed unfit for, or who fail to respond to, loco-regional therapies [4,5]. In field practice studies which have assessed the efficacy and cost-effectiveness of Sorafenib for HCC, high rates of treatment discontinuation have occurred, due to tumor progression, liver decompensation, and severe adverse events (SAEs) [6,7,8,9,10].

Until 2018, Sorafenib was the only systemic treatment which was found to significantly improve overall survival (OS) as a first-line therapy for advanced HCC [11]. Subsequently, Lenvatinib was found to be non-inferior to Sorafenib in terms of OS, and it also exhibited significantly better progression-free survival (PFS) in a randomized controlled trial (RCT) [12]. More recently, immune checkpoint inhibitors (ICIs), particularly antibodies targeting the programmed cell death-1 (PD-1)/programmed cell death ligand-1 (PD-L1) pathway, have been a major breakthrough in drug development for HCC [13]. In 2020, combined therapy with Atezolizumab and Bevacizumab has exhibited a significant improvement in OS and PFS compared with Sorafenib [14].

Among patients who have progressed and are tolerant to Sorafenib, treatment with Regorafenib has resulted in longer OS than placebo [15]. Thus, Regorafenib has become the first second-line drug to be approved for HCC [15]. Subsequently, Cabozantinib [16] and Ramucirumab [17] have exhibited a survival benefit versus placebo in second-line phase III RCTs. For Ramucirumab, these results were observed in patients with elevated alpha-fetoprotein. After 2017, the Food and Drug Administration granted accelerated approval to Nivolumab and Pembrolizumab, two monoclonal antibodies which act as inhibitors of PD-1 in a second-line setting [18,19,20,21,22]. More recently, however, Pembrolizumab failed to achieve statistically significant improvement in OS and PFS compared to placebo in second-line setting [23].

To date, the best sequential first- and second-line systemic treatment remains elusive. In particular, what is the best sequential systemic strategy in terms of OS benefit and safety? Due to the lack of sequential appropriately designed RCTs, these issues remain unanswered.

Therefore, we have developed a Markov model based on data from available RCTs, and we describe its use to evaluate the risks and benefits of currently available sequential systemic treatments for patients with advanced HCC.

## 2. Results

**Figure 1:**
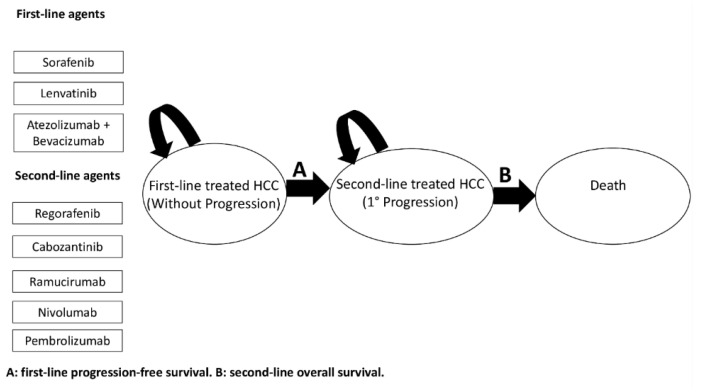
General structure of the Markov model used.

**Figure 2:**
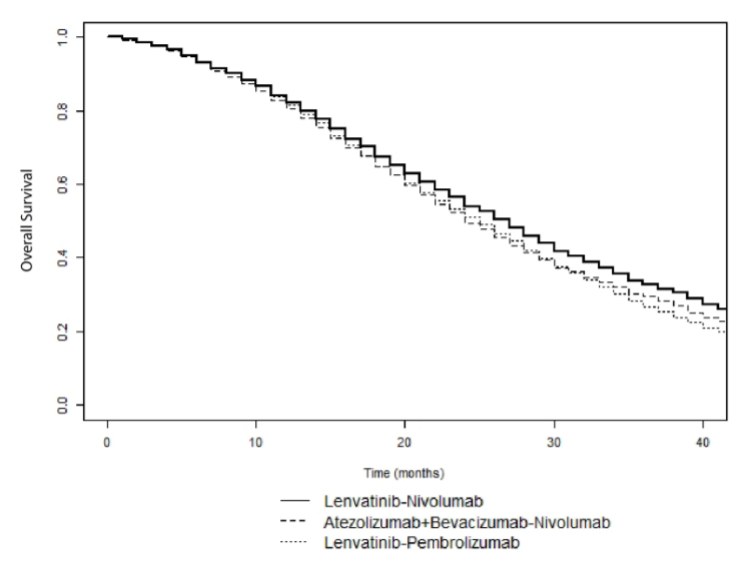
Simulated survival curves of Lenvatinib followed by Nivolumab, Lenvatinib followed by Pembrolizumab, and Atezolizumab plus Bevacizumab followed by Nivolumab sequences in patients with advanced HCC.

The general structure of the Markov model is showed in Figure 1. Outcomes reported in the clinical trials examined and model parameters are reported in Table 1 and Appendix A Table A1 and Table A2. The base-case effectiveness and safety of the 15 treatment sequences ordered according to median OS are reported in Table 2. First-line Lenvatinib followed by second-line Nivolumab was the most effective treatment (median OS, 27 months), with a LYG value of 0.75 and a NNT of 5. In contrast, first-line Sorafenib followed by second-line Ramucirumab was the least effective treatment sequence (median OS, 18 months). Figure 2 shows the simulated survival curves of the three most effective treatment sequences (Lenvatinib followed by Nivolumab, Lenvatinib followed by Pembrolizumab, and Atezolizumab plus Bevacizumab followed by Nivolumab, respectively). At a threshold of 1000 patients per sequence, the simulated estimates of median OS were significantly higher for Lenvatinib followed by Nivolumab, compared to Lenvatinib followed by Pembrolizumab (*p* = 0.002) and Atezolizumab plus Bevacizumab followed by Nivolumab (*p* = 0.035) (Supplementary Table S1). Survival curves for the other sequences are shown in Supplementary Figures S1–S3.

Among the 15 treatment sequences analyzed, the treatment sequence with the lowest rate of SAEs was first-line Atezolizumab plus Bevacizumab followed by second-line Nivolumab (40.2%). Conversely, the treatment sequences exhibiting the highest toxicity (77.2% SAEs) were first-line Lenvatinib followed by second-line Cabozantinib or Regorafenib. (Table 2).

**Figure 3:**
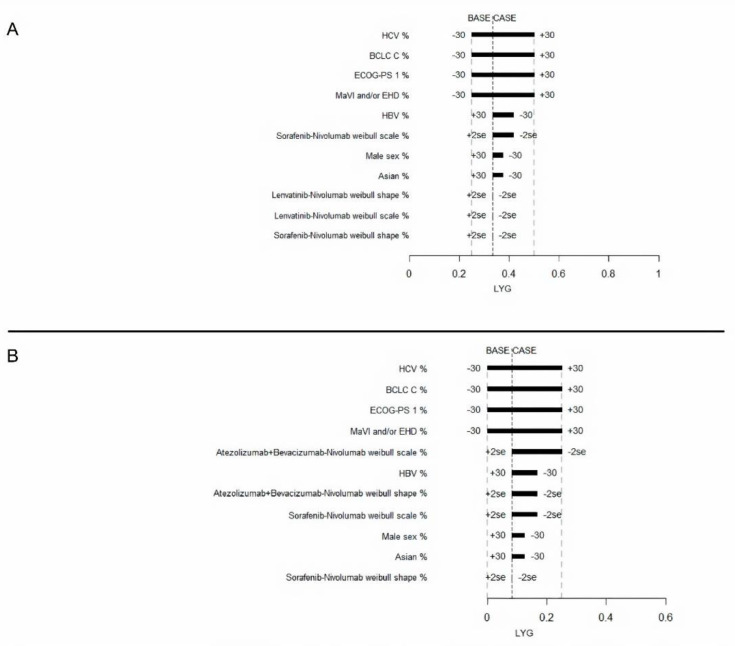
Tornado diagram for the one-way sensitivity analysis of variables influencing LYG of advanced HCC patients treated with Lenvatinib-Nivolumab (**A**) or Atezolizumab plus Bevacizumab-Nivolumab (**B**). Both were compared to the Sorafenib-Nivolumab treatment.

Next, the most effective sequence (Lenvatinib followed by Nivolumab) was compared with the safest sequence (Atezolizumab plus Bevacizumab followed by Nivolumab). A one-way sensitivity analysis for LYG was performed for these two sequences in comparison with Sorafenib followed by Nivolumab. LYG ranged from 0.25 to 0.50 for Lenvatinib followed by Nivolumab, and from 0 to 0.25 for Atezolizumab plus Bevacizumab followed by Nivolumab. The most influential factors identified for both strategies were: HBV infection, HCV infection, MaVI and/or EHD, ECOG-PS 1, and BCLC stage C (Figure 3). Variations of ±30% in the distribution of these variables had a greater influence on their effectiveness.

Compared to Atezolizumab plus Bevacizumab followed by Nivolumab, the ISER of Lenvatinib followed by Nivolumab was 32% of SAEs for LYG. One-way sensitivity analysis of the ISER also showed that MaVI and/or EHD was the most influential factor, producing an ISER ranging from 32–94% of SAEs for LYG. (Supplementary Figure S4).

**Figure 4:**
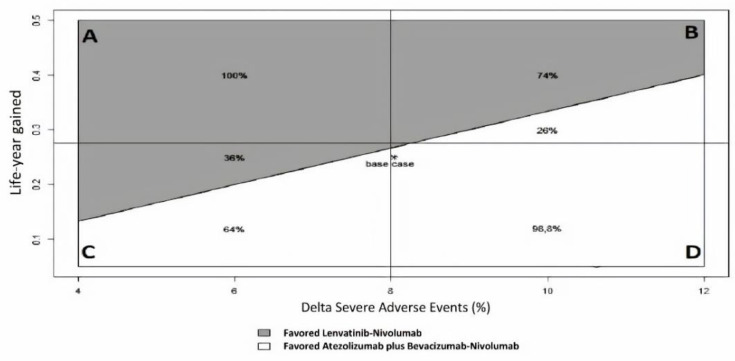
Two-way sensitivity analysis of ISER (Delta Severe Adverse Events%/Life-year gained) to indicate which therapy is favored for varying Severe Adverse Events (SAEs) and LYG (Life-Year Gained) values, according to a “willingness-to-risk” threshold of 30% of SAEs for LYG. Grey area favored Lenvatinib-Nivolumab, while white area favored Atezolizumab plus Bevacizumab-Nivolumab. Four clinical scenarios were modeled: (**A**) high LYG, low SAEs; (**B**) high LYG, high SAEs; (**C**) low LYG, low SAEs; (**D**) low LYG, high SAEs.

A two-way sensitivity analysis was performed to demonstrate which therapy would be preferred for varying the difference in SAE and LYG values (Figure 4 and Supplementary Figure S5). At a willingness-to-risk threshold of 30% of SAEs for LYG, first-line Lenvatinib followed by second-line Nivolumab was favored in 52% of cases overall. For instance, in scenario B (high LYG and high SAEs), Lenvatinib followed by Nivolumab was favored in 74% of cases, while in scenario C (low LYG and low SAEs), it was favored in only 36% of cases.

The results of a probabilistic analysis for LYG are presented in Figure S6. In the Monte Carlo simulation, the varying prevalence of HBV infection, HCV infection, MaVI and/or EHD, and ECOG-PS 1 simultaneously, Lenvatinib followed by Nivolumab, and Atezolizumab plus Bevacizumab followed by Nivolumab, were more effective than Sorafenib followed by Nivolumab in 100% and 92% of the simulations, respectively.

## 3. Discussion

It is well known that sequential trials in oncology are difficult to perform, and in most settings, such as advanced HCC, they are practically unfeasible. Moreover, real-world data of sequential therapies in advanced HCC are time consuming and they are lacking to date. Therefore, comparative evidence among different treatment sequences could be obtained only by simulation models. In the field of HCC, more innovative treatments are found to confer significant survival benefits, even when used in the advanced stages of disease. To our knowledge, this is the first model to date which provides a comprehensive comparison of different sequential strategies for patients with advanced or intermediate HCC, who are unfit for, or who do not respond to, loco-regional therapies.

Our model suggests that first-line Lenvatinib followed by second-line Nivolumab or Pembrolizumab may be an attractive sequential systemic strategy in terms of OS for patients with advanced HCC. Meanwhile, first-line Atezolizumab plus Bevacizumab followed by second-line Nivolumab was identified as the safest sequential strategy. Strategies involving first-line MKI followed by second-line MKI appear to be associated with worse survival in all scenarios. Using a willingness-to-risk threshold of 30% of SAEs for LYG, the net health benefit assessed by ISER favored Lenvatinib followed by Nivolumab compared to Atezolizumab plus Bevacizumab, followed by Nivolumab in 52% of cases, giving a wide range of uncertainty about the net health benefit between the two compared sequences.

RCTs of systemic therapies for advanced HCC have often showed small, albeit significant, differences in OS between active drug and placebo. If the OS gain is relatively small (on the order of a few months), the quality of life spent on or off active treatment is crucial, and it should be considered in the clinical decision-making process. Hence, our model provides evidence that first- and second-line ICIs are the safest sequential strategies. Moreover, sequential first-line ICIs should be reserved for the most fragile patients, considering the relatively improved safety profile they exhibit compared to MKIs. However, ICIs are associated with immune-mediated hepatotoxicity whose severity is unpredictable. The hepatotoxicity can range from mild to life-threatening, and it is not completely known in patients with cirrhosis [29,30].

According to ASCO statements [31], net health benefit is defined as the balance between clinical benefit and SAEs, and it is used to assess the magnitude of the difference between two different therapeutic strategies [32]. In this model, we proposed an innovative measure that combines effectiveness and safety, called ISER. Given the wide range of uncertainty on the net health benefit assessed by ISER between Lenvatinib followed by Nivolumab compared to Atezolizumab plus Bevacizumab followed by Nivolumab, the best treatment strategy should be carefully agreed upon, with the policy makers also taking into account different factors, including the cost-effectiveness.

The results obtained in the present study appear to be plausible and are supported by other evidence. A study of tumor microenvironment and immune setting in patients treated with Sorafenib has revealed a possible role for MKIs also in immunomodulation [33]. Furthermore, Sorafenib treatment may increase the expression of PD-L1 in tumor-infiltrating immune cells [33]. While the expression of PD-L1 in tumor microenvironments has been evaluated as a predictive biomarker for immunotherapy in other solid tumors (i.e., melanoma, non-small cell lung cancer), the CheckMate 040 trial assessing Nivolumab as a second-line treatment for HCC did not find any differences in cancer response according to PD-L1 expression in tumor cells [19]. However, PD-L1 expression was not assessed in tumor-infiltrating immune cells which are involved in immune-surveillance, and probably in HCC progression, after Sorafenib treatment. Additional evidence involves a potential synergistic role for Lenvatinib with ICIs, thereby inhibiting an immunosuppressive microenvironment and promoting anti-tumor immune activation [34]. Mounting evidence from preclinical and clinical studies supports the use of immunotherapy as a second-line treatment after locoregional treatments, such as thermoablation, to increase the antitumor immune response [35]. It has been proposed that hyperthermia may activate quiescent dendritic cells and improve leukocyte trafficking, thereby enhancing the effectiveness of subsequent immunotherapy. Finally, it should be considered that only few data exist in the Imbrave 150 [14] and in KeyNote 240 [23] RCTs regarding the sequential treatment of first-line ICIs followed by second-line ICIs, and that it may not have a strong biological plausibility. However, the sequence ICIs-ICIs was used in other oncological settings, i.e., Ipilimumab followed by Nivolumab was used in patients with advanced melanoma [36,37].

There are several limitations associated with our model.

(1) The biggest and most important issue of our decision model is that it did not consider the carry-over effect. Further sequential trials with a crossover design, properly designed to assess the carryover effect, could solve this intriguing issue.
(2) The lack of clinical data concerning the majority of sequential strategies assessed by our model. All the five available second-line therapies were approved in trials, including Sorafenib-experienced patients, while no second-line therapy was evaluated after Lenvatinib or Atezolizumab plus Bevacizumab. As Atezolizumab plus Bevacizumab will become the new standard first-line treatment of advanced HCC, the identification of the optimal treatment sequential strategy represents a burning unsolved medical need. Therefore, our decision model, with all the limitations of a simulation, should be considered a useful tool to evaluate the risk-benefit ratio of treatment sequences not evaluable in the trials performed to date. In the absence of sequential RCTs, large-scale real-world studies are urgently needed.
(3) Trials of second-line treatment included in the model had some differences in inclusion and exclusion criteria, particularly regarding MKIs. However, we are confident that the patients enrolled in second-line RCTs that we examined could be roughly comparable, because all of the patients were Sorafenib-experienced.
(4) Assessment of the benefit of ICIs raises several issues considering the unconventional pattern of radiological response or progression. Novel radiological response assessment tools (i.e., immuneRECIST) and innovative methodologies (i.e., Weibull distribution, restricted mean survival time, landmark analyses) may be able to capture the long-term benefit observed with ICIs. In addition, the use of multivariate meta-analysis, which combines surrogate endpoints (i.e., PFS and objective response rate) with OS, may prove useful for substantiating the benefit of different sequential treatments on OS.
(5) Lack of data regarding all-cause SAEs for Nivolumab may have affected our analyses. However, reported data for Pembrolizumab led to a more accurate assessment of overall tolerability of ICIs in patients with liver disease [30].
(6) Unfortunately, we were unable to calculate quality-adjusted life-years (QALYs), since utilities were not available in the majority of the RCTs examined, particularly regarding ICI trials. Accordingly, we assessed LYG as the primary measure of efficacy. However, utilities may vary widely across different patient subgroups, and they critically depend on quality of life assumptions.
(7) Lack of data on potentially relevant prognostic factors (i.e., microscopic vascular invasion, histological grading, gene profiling [2]), and liver decompensation, that is, a well-known driver of death in HCC patients [38,39], may have affected our results.

The main strengths of our study are that: (1) this is the first model to estimate survival in sequential therapies by using PFS for first-line therapy and OS for second-line therapy. Simulation remains today the only method to provide quantitative estimates for physicians and policymakers in the setting of advanced HCC; (2) it provides a novel method to estimate the net health benefit by the use of the innovative measure, so-called ISER.

## 4. Materials and Methods

### 4.1. Decision Model

A semi-Markov model was developed to analyze the effectiveness of various sequential systemic treatments for advanced HCC over a lifetime horizon (Figure 1), and to allow the transition hazard to increase over time. This approach is parametric, and it was performed by estimating the scale and shape parameter of a Weibull distribution. Natural mortality rate at age of population was included in the model for a sixty-year-old hypothetical male patient. Given the very short life expectancy, we did not discount risks and benefits. The sequential semi-Markov model was built by using an ad hoc routine written with R statistical software. We simulated the clinical history of a hypothetical cohort of patients with advanced HCC, which had the same characteristics as patients enrolled in clinical trials [12,14,15,16,20,23,24,25,26,27,28] (see Table 1 and Table A1). PFS was used as the main endpoint for first-line treatments, because it is not influenced by post-progression survival, and because it is a validated surrogate for OS in advanced HCC [40]. In addition, data describing OS and PFS treatment benefits were derived from Kaplan-Meier curves from the above-mentioned trials. In particular, PFS curves of patients receiving Sorafenib as a first-line treatment were extracted from reports of six phase III RCTs [12,14,24,25,26,27]. For Lenvatinib and Atezolizumab plus Bevacizumab, PFS curves were extracted from the REFLECT trial [12] and the IMbrave150 trial [14], respectively. OS curves for patients treated with second-line therapies were extracted from RESORCE [15], CELESTIAL [16], REACH [28], CheckMate-040 [20], and KeyNote-240 [23] trials. Individual patient survival data were reconstructed by using an algorithm proposed by Guyot et al. [41] This algorithm provides a list of patients with predicted survival times and a predicted event of interest (i.e., alive or dead; progression or no progression) by using digitalized data on survival probabilities, time, and total number of patients and events. Engauge Digitizer software Version 12 was used to extract data from the curves. Each reconstructed survival curve was inspected for accuracy and was compared with originally published curves. Supplementary Figures S7–S14 show the Kaplan–Meier curves which were reconstructed from published curves, with estimated Weibull survival curves superimposed. The scale and shape parameter of a Weibull distribution on the data extracted from Kaplan-Meier curves are showed in Table A2. The impact of alternative survival distributions (lognormal, log-logistic, exponential, gamma, generalized gamma) on predicted survival probability was also explored, although none of the considered distributions resulted in a suitable alternative to the Weibull distribution. The lognormal, log-logistic, and exponential distributions were excluded a priori, because they do not allow monotonically increasing hazards to fit, which is a fundamental assumption for the type of disease being evaluated. Similarly, although the gamma and generalized gamma distributions could theoretically allow monotonically increasing hazards, these were first fitted and then excluded after a visual inspection, which showed decreasing (gamma) or dome-shaped (generalized gamma) hazards. *p*-values ≤ 0.05 were considered statistically significant. As a safety measure, the number of SAEs (grade ≥ 3) from each included trial was extracted. The model evaluated 15 sequential therapy scenarios (Figure S15).

### 4.2. Model Transitions and Survival Estimates

The model simulated transitions among the following three health states, with a cycle length of one month: (1) advanced HCC eligible to first-line systemic therapy; (2) first progression; and (3) death (Figure 1). The health states were mutually exclusive (i.e., a patient could experience a single health state at any given time). Survival estimates for sequential settings considered the proportion of patients who did not receive second-line therapy due to death during first-line therapy. Furthermore, transitions of patients between health states were based on calculated transition probabilities from PFS and OS data extracted from published studies. Simulation of 2000 pseudo-random patients was performed to obtain overall simulated survival times and median times throughout the three states of the disease.

### 4.3. Outcomes

Life-year gained (LYG) was the main health outcome. Median OS obtained from the Markov model at different simulated sample sizes was compared by using the Wilcoxon test, in order to identify the minimum sample size needed to assess a statistically significant difference. To provide long-term survival information, a milestone survival analysis at 36 months was performed. Accordingly, the number needed to treat (NNT) was calculated for each sequential strategy.

The rate of SAEs (≥grade 3) was calculated for each sequential treatment, by taking into account transition probabilities from first- to second-line treatments, by using a weighted mean of the number of simulated patients transited in each disease state (initial treatment and cancer progression). To obtain a net health benefit measure [31], clinical benefit and toxicity were combined to calculate a novel measure, incremental safety-effectiveness ratio (ISER). The latter was defined as the difference in the rate of SAEs between two sequential treatments, divided by their difference in effectiveness, and measured in LYG. This unit of measure expresses the incremental percentage of SAEs for each LYG.

$$ISER=\frac{Delta\;SAEs\%\;}{LYG}$$

### 4.4. Deterministic Sensitivity Analysis

Two different deterministic sensitivity analyses were performed for LYG and ISER. The following variables were evaluated: male gender, Asian ethnicity, hepatitis C virus (HCV) and hepatitis B virus (HBV) as etiology of liver disease, macrovascular invasion (MaVI) and/or extrahepatic disease (EHD), Eastern Cooperative Oncology Group (ECOG) Performance Status 1, and Barcelona Clinic Liver Cancer (BCLC) stage C. (see Appendix A Table A2) We considered ± two times the standard error (SE) to be the variation range for both the Weibull scale and shape parameters. We considered ±30% variation for the remaining variables. The latter was actually corrected for the maximum possible variation (i.e., a 30% increase in MAVI was impossible, as the baseline value was 88%, so a 12% increase was considered, up to 100% of MAVI).

In the one-way deterministic sensitivity analysis for LYG, Lenvatinib followed by Nivolumab and Atezolizumab plus Bevacizumab followed by Nivolumab were indirectly compared to Sorafenib followed by Nivolumab.

In the one-way deterministic sensitivity analysis for ISER, Lenvatinib followed by Nivolumab versus Atezolizumab plus Bevacizumab followed by Nivolumab was compared, because the latter dominates Sorafenib followed by Nivolumab, both in terms of median survival and SAEs.

Two-way sensitivity analysis for ISER was performed by varying the numerator (percentage difference in SAEs) and the denominator (LYG). The variation range for the denominator was chosen to be as large as the maximal value observed, while a ±4% variation was chosen for the numerator. Analogous to the willingness-to-pay aspect of a cost-effectiveness analysis, a willingness-to-risk threshold value must also be established for ISER. Several possible willingness-to-risk thresholds for ISER, and their visual effect in terms of estimated areas, when SAEs and LYG are varied according to their ranges, have been calculated.

### 4.5. Probabilistic Sensitivity Analysis

To examine the extent to which differences in LYG could be explained by differences in patient characteristics, a two-step probabilistic sensitivity analysis was performed. In the first step, four variables (i.e., proportion of patients with HCV infection, HBV infection, ECOG—Performance Status 1, and MacroVascular Invasion and/or extrahepatic disease) were included in a meta-regression model. The dependent variable was median PFS time. The meta-regression model employed was a weighted multiple linear regression model, where the weight was the study sample sizes. By assuming a uniform distribution for the values of the four variables considered, 1000 pseudo-random values were generated from each variable, in order to obtain corresponding predictions from the model. In the second step, the predicted median times were then proportionally translated into Weibull scale parameters by assuming, in percentage, a proportional shift of the scale parameters for the base case (hazard proportionality is a property of the Weibull distribution). Then, in turns, these parameters were used as inputs in the Markov model, to obtain 1000 simulated median times.

## 5. Conclusions

Single-agent first-line Lenvatinib, followed by second-line Nivolumab or Pembrolizumab, seemed to be an attractive option for advanced HCC. Meanwhile, the first-line combination of Atezolizumab plus Bevacizumab, followed by second-line Nivolumab, was the safest sequential strategy. Congruent with the above data, a wide range of uncertainty remains about what is the optimal sequential strategy in terms of net health benefit. Ongoing and future RCTs and data obtained in field practice studies will confirm whether combination or sequential systemic therapies are more beneficial. In the meantime, clinicians, patients and policymakers can use this decision model for the collaborative decision-making process.

## Figures and Tables

**Table 1:** Outcomes reported in the clinical trials examined and model parameters.

Variables	Base-Case	References
**First-line systemic treatments**		
Median PFS (months) reported in RCTs		
Sorafenib	3.8 mo	[12,14,24,25,26,27]
Lenvatinib	7.4 mo	[12]
Atezolizumab plus Bevacizumab	6.8 mo	[14]
Median PFS (IQR) (months) obtained with Weibull distribution		
Sorafenib	4.6 (6.7) mo	
Lenvatinib	8.8 (11.7) mo	
Atezolizumab plus Bevacizumab	7.2 (9.3) mo	
SAEs		
Sorafenib	72.3%	[12,14,24,25]
Lenvatinib	75%	[12]
Atezolizumab plus Bevacizumab	61%	[14]
**Second-line systemic treatments**		
Median OS (months) reported in RCTs		
Regorafenib	10.6 mo.	[15]
Cabozantinib	10.2 mo	[16]
Ramucirumab	9.2 mo	[28]
Nivolumab	15.1 mo	[20]
Pembrolizumab	13.9 mo	[23]
Median OS (IQR) (months) obtained with Weibull distribution		
Regorafenib	11.4 (14.4) mo	
Cabozantinib	11.4 (14) mo	
Ramucirumab	11.3 (14.5) mo	
Nivolumab	16.4 (23.2) mo	
Pembrolizumab	14 (17.8) mo	
SAEs		
Regorafenib	79.7%	[15]
Cabozantinib	79.4%	[16]
Ramucirumab	70.4%	[28]
Nivolumab	18.6% *	[20]
Pembrolizumab	52.7%	[23]

OS, overall survival. PFS, Progression-free survival. mo, months. IQR, Interquartile Range. SD, Standard Deviation. SAEs, severe adverse events. BCLC, Barcelona Clinic Liver Cancer. RCTs, randomized controlled trials. For Sorafenib, data were pooled from the available RCTs [14,16,24,25,26,27]. * Because the incidence of treatment emergent AEs was not reported for Nivolumab, we reported severe treatment-related adverse events.

**Table 2:** Base-case effectiveness and safety of 15 treatment sequences according to median OS.

Treatment Sequence	Median OS (mo)	NNT	36-Month OS (%)	LYG (yr)	SAEs (%)
Lenvatinib-Nivolumab	27	5	32.9	0.75	48.0
Lenvatinib-Pembrolizumab	25	7	26.8	0.58	64.4
Atezolizumab plus Bevacizumab-Nivolumab	24	6	29.5	0.50	40.2
Sorafenib-Nivolumab	23	7	26.1	0.42	46.3
Atezolizumab plus Bevacizumab-Pembrolizumab	23	9	23.4	0.42	57.1
Lenvatinib-Ramucirumab	22	13	19.9	0.33	72.8
Lenvatinib-Regorafenib	22	14	19.3	0.33	77.2
Lenvatinib-Cabozantinib	22	14	19.2	0.33	77.2
Sorafenib-Pembrolizumab	20	15	18.9	0.17	62.9
Atezolizumab plus Bevacizumab-Ramucirumab	20	23	16.7	0.17	65.6
Atezolizumab plus Bevacizumab-Regorafenib	20	29	15.8	0.17	70.0
Atezolizumab plus Bevacizumab-Cabozantinib	20	30	15.6	0.17	69.9
Sorafenib-Cabozantinib	18	83	11.1	0.00	75.8
Sorafenib-Regorafenib	18	200	11.8	0.00	75.9
Sorafenib-Ramucirumab	18	-	12.3	0.00	71.4

LYG, life-year gained. All the sequences were compared to the worst sequence (Sorafenib-Ramucirumab). OS, overall survival. SAEs, severe adverse events. NNT, number needed to treat. yr, years. mo, months.

## Supplementary Materials

The following are available online at https://www.mdpi.com/2072-6694/12/8/2132/s1, Figure S1. Simulated survival curves of sequential systemic treatment strategies in patients with advanced hepatocellular carcinoma (first-line Sorafenib); Figure S2. Simulated survival curves of sequential systemic treatment strategies in patients with advanced hepatocellular carcinoma (first-line Lenvatinib); Figure S3. Simulated survival curves of sequential systemic treatment strategies in patients with advanced hepatocellular carcinoma (first-line Atezolizumab plus Bevacizumab); Figure S4. Tornado diagram for a one-way sensitivity analysis of variables which influenced the incremental safety-effectiveness ratio (ISER) of Lenvatinib followed by Nivolumab compared to Atezolizumab + Bevacizuab followed by Nivolumab in patients with advanced hepatocellular carcinoma; Figure S5. Contour plot of ISER for varying delta SAE and LYG values; Figure S6. Empirical cumulative density function curves representing the probability that Lenvatinib followed by Nivolumab or Atezolizumab plus Bevacizuab followed by Nivolumab are more effective than Sorafenib followed by Nivolumab in patients with advanced hepatocellular carcinoma Figure S7: Kaplan-Meier curve with Weibull estimated curve for Sorafenib treatment arms superimposed; Figure S8: Kaplan-Meier curve with Weibull estimated curve for Lenvatinib superimposed; Figure S9. Kaplan-Meier curve with Weibull estimated curve for Atezolizumab + Bevacizumab superimposed; Figure S10. Kaplan-Meier curve with Weibull estimated curve for Regorafenib superimposed; Figure S11. Kaplan-Meier curve with Weibull estimated curve for Cabozantinib superimposed; Figure S12. Kaplan-Meier curve with Weibull estimated curve for Ramucirumab superimposed; Figure S13. Kaplan-Meier curve with Weibull estimated curve for Nivolumab superimposed; Figure S14. Kaplan-Meier curve with Weibull estimated curve for Pembrolizumab superimposed; Figure S15. Fifteen sequential systemic treatment strategies deriving from three first-line and five second-line treatments for patients with advanced HCC; Table S1. Comparison of median overall survival (OS) between Lenvatinib followed by Nivolumab and Atezolizumab + Bevacizumab followed by Nivolumab. The simulated sample size (N) was varied for each treatment arm

## Appendix A

**Table A1:** Characteristics of the clinical trials from which model data were extracted.

Trial	Treatments	N of Patients in Each Arm	Characteristics of Included Patients	Median OS (mo.)	Median PFS (mo.)	Median TTP (mo.)	ORR (%)	SAEs (%)
**First-line**								
REFLECT [12]	Sorafenib; Lenvatinib	476;478	Unresectable HCC BCLC B or C; Child-Pugh A; ECOG-PS 0 or 1; one or more measurable target lesions. Patients with 50% or higher liver occupation, invasion of the bile duct, or invasion at the main portal vein and patients previously treated with systemic therapy were excluded.	12.3; 13.6	3.7; 7.4	3.7; 8.9	12.4; 40.6	66.5; 75
IMBrave150 [14]	Sorafenib; Atezolizumab+Bevacizumab	165;336	Locally advanced or metastatic and/or unresectable HCC; No prior systemic therapy.	13.2; NE	4.3; 6.8	-	12; 27	60.9; 61
SUN1170 [35]	Sorafenib; Sunitinib	544;530	Histologically confirmed, locally advanced or metastatic HCC candidate for systemic anticancer treatment. Child-Pugh A; ECOG-PS 0 or 1	10.2; 7.9	3.0; 3.6	3.8; 4.1	6.1; 6.6	74.2; 82.1
SIRveNIB [36]	Sorafenib;Y90	178;182	Locally advanced cancer (BCLC) stage B or C without extrahepatic disease with or without portal vein thrombosis, not amenable to curative treatment modalities. No more than 2 previous hepatic artery–directed treatments.	10.0; 8.8	5.1; 5.8	5.4; 6.1	-	50.6; 27.7
SILIUS [37]	Sorafenib; HAIC	103;103	Advanced HCC (four or more tumours refractory to transarterial chemoembolisation or tumours with vascular invasion or extrahepatic spread) not candidates for hepatectomy, local ablation or transarterial chemoembolisation; Child-Pugh A-B7; ECOG-PS 0-1	11.5; 11.8	3.5; 4.8	3.5; 5.3	18; 36	-
SARAH [38]	Sorafenib; Y90	222;459	Locally advanced HCC BCLC C or new HCC not eligible for surgical resection, liver transplantation, or thermal ablation after a previously cured HCC (cured by surgery or thermoablative therapy), or HCC with two unsuccessful rounds of transarterial chemoembolization: Child-Pugh A-B7; ECOG-PS 0-1	9.9; 8.0	3.7; 4.1	-	12; 19	-
**Second-line**								
RESORCE [15]	Regorafenib; Placebo	379;194	HCC BCLC B or C not amenable to curative treatment; Child-Pugh A; ECOG-PS 0 or 1; documented radiological progression during Sorafenib treatment; tolerance to Sorafenib (≥400 mg daily for at least 20 of the 28 days before discontinuation); last Sorafenib dose within 10 weeks of randomization. Patients with previous systemic treatment for HCC or discontinuation of Sorafenib for toxicity were excluded.	10.6; 7.8	3.1; 1.5	3.2; 1.5	11; 4	79.7; 58.5
CELESTIAL [16]	Cabozantinib; Placebo	470;237	HCC not amenable to curative Treatment; Child-Pugh class A; ECOG-PS 0 or 1 previous treatment with Sorafenib; disease progression after at least one systemic treatments (up to 2)	10.2; 8.0	5.2; 1.9	-	4; 1	79.4; 48.1
REACH [39]	Ramucirumab; Placebo	283;282	BCLC C or B refractory or not amenable to locoregional therapy; Child-Pugh A; ECOG-PS 0 or 1; previous treatment with Sorafenib (stopped because of progression or intolerance).	9.2; 7.6	2.8; 2.1	3.5; 2.6	7; <1	70.4; 54.3
CheckMate 040 [19,20]	Nivolumab	214	Histologically confirmed advanced HCC (not amenable to curative surgery or local treatment); Child-Pugh A or B7; ECOG-PS 0 or 1; disease progression while receiving at least one previous line of systemic therapy, including sorafenib or intolerance to Sorafenib.	15.1	-	-	14	18.6
KeyNote-240 [23]	Pembrolizumab; Placebo	278;135	BCLC C or B refractory or not amenable to locoregional therapy; Child-Pugh A; ECOG-PS 0 or 1; radiographic progression during or intolerance to sorafenib treatment	13.9; 11.6	3.0; 2.8	3.8; 2.8	18.3; 4.4	52.7; 46.3

HCC, Hepatocellular carcinoma. BCLC, Barcelona Clinic Liver Cancer. PS, Performance status. OS, Overall Survival. PFS, Progression-Free Survival. TTP, Time To Progression. ORR, Objective Response Rate. Mo., months. SAEs, Severe (grade ≥ 3) adverse events.

**Table A2:** Model parameters and characteristics of patients.

Variables	Base-Case	References
**First-line systemic treatments**		
Weibull distribution parameters of PFS (scale; shape)		
Sorafenib	6.48; 1.09	
Lenvatinib	12.06; 1.18	
Atezolizumab plus Bevacizumab	9.78; 1.2	
Mean PFS (SD) (months) obtained with Weibull distribution		
Sorafenib	6.5 (0.13) mo.	
Lenvatinib	10.8 (0.44) mo.	
Atezolizumab plus Bevacizumab	9.1 (0.43) mo.	
Characteristics of patients		
Male sex		
Sorafenib	85.5%	[12,14,35,36,37,38]
Lenvatinib	84.7%	[12]
Atezolizumab plus Bevacizumab	82.4%	[14]
Asian ethnicity		
Sorafenib	64.6%	[12,14,35,36,37,38]
Lenvatinib	69.9%	[12]
Atezolizumab plus Bevacizumab	39.6%	[14]
HBV		
Sorafenib	39.7%	[12,14,35,36,37,38]
Lenvatinib	52.5%	[12]
Atezolizumab plus Bevacizumab	48.8%	[14]
HCV		
Sorafenib	23.4%	[12,14,35,36,37,38]
Lenvatinib	19.0%	[12]
Atezolizumab plus Bevacizumab	21.4%	[14]
Vascular Invasion and/or extrahepatic disease		
Sorafenib	63.3%	[12,14,35,36,37,38]
Lenvatinib	68.8%	[12]
Atezolizumab plus Bevacizumab	76.8%	[14]
ECOG Performance status 1		
Sorafenib	32.0%	[12,14,35,36,37,38]
Lenvatinib	36.4%	[12]
Atezolizumab plus Bevacizumab	37.7%	[14]
BCLC C		
Sorafenib	71.4%	[12,14,35,36,37,38]
Lenvatinib	78.2%	[12]
Atezolizumab plus Bevacizumab	82.1%	[14]
**Second-line systemic treatments**		
Weibull distribution parameters (scale; shape)		
Regorafenib	15.36; 1.23	
Cabozantinib	15.24; 1.27	
Ramucirumab	15.29; 1.21	
Nivolumab	22.8; 1.11	
Pembrolizumab	18.96; 1.22	
Mean OS (SD) (months) obtained with Weibull distribution		
Regorafenib	14.8 (0.62) mo.	
Cabozantinib	14.7 (0.54) mo.	
Ramucirumab	14.9 (0.7) mo.	
Nivolumab	22.2 (1.34) mo.	
Pembrolizumab	18.5 (0.87) mo.	
SAEs		
Regorafenib	79.7%	[15]
Cabozantinib	79.4%	[16]
Ramucirumab	70.4%	[39]
Nivolumab	18.6% *	[20]
Pembrolizumab	52.7%	[23]

HCC, Hepatocellular carcinoma. BCLC, Barcelona Clinic Liver Cancer. PS, Performance status. OS, Overall Survival. PFS, Progression-Free Survival. Mo., months. SAEs, Severe (grade ≥ 3) adverse events.
