# Supplementary material for: Optimizing Sequential Systemic Therapies for Advanced Hepatocellular Carcinoma: A Decision Analysis
Source: Cancers (Basel). 2020 Jul 31;12(8):2132. doi: 10.3390/cancers12082132 (PMC7464739; doi:10.3390/cancers12082132)
Supplement: Supplementary file 1 [file cancers-12-02132-s001.pdf]

cancers

MDPI

Supplementary Materials

Optimizing Sequential Systemic Therapies for Advanced Hepatocellular Carcinoma: A Decision Analysis.

Giuseppe Cabibbo, Ciro Celsa, Marco Enea, Salvatore Battaglia, Giacomo Emanuele Maria Rizzo, Stefania Grimaudo, Domenica Matranga, Massimo Attanasio, Paolo Bruzzi, Antonio Craxì and Calogero Cammà

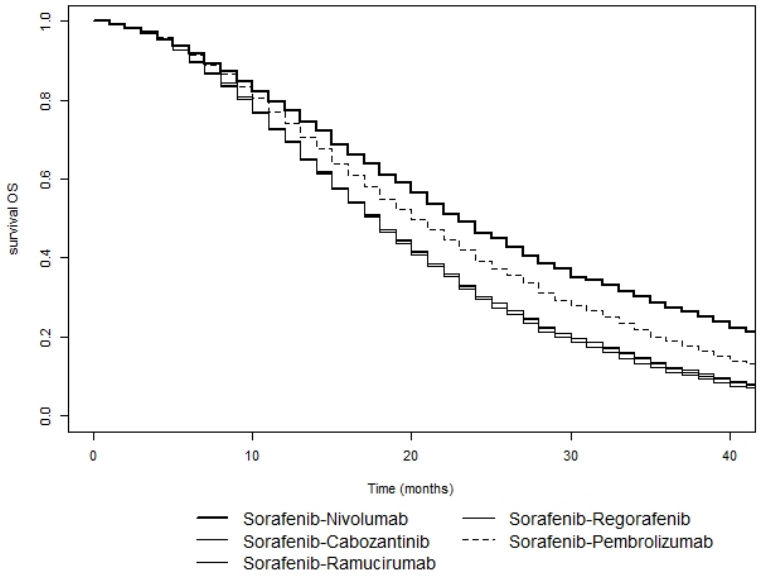

Figure S1. Simulated survival curves of sequential systemic treatment strategies in patients with advanced hepatocellular carcinoma (first-line Sorafenib).

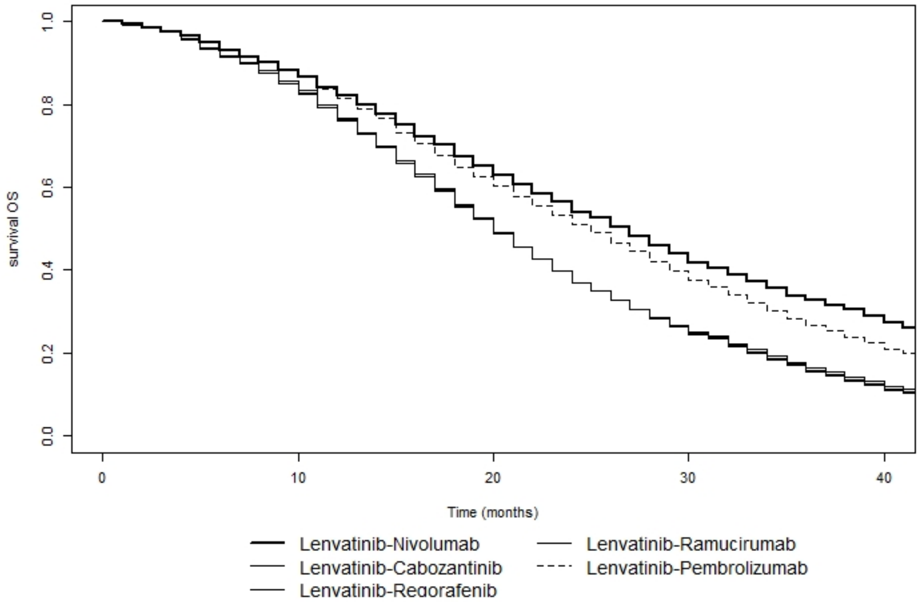

Figure S2. Simulated survival curves of sequential systemic treatment strategies in patients with advanced hepatocellular carcinoma (first-line Lenvatinib).

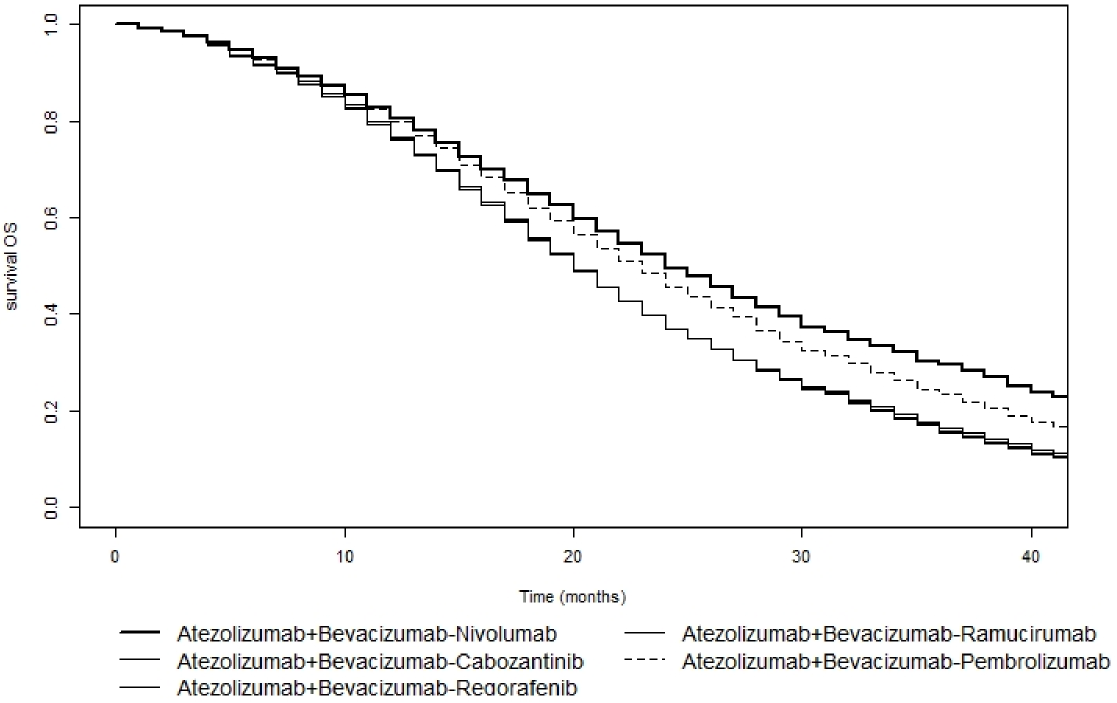

Figure S3. Simulated survival curves of sequential systemic treatment strategies in patients with advanced hepatocellular carcinoma (first-line Atezolizumab plus Bevacizumab).

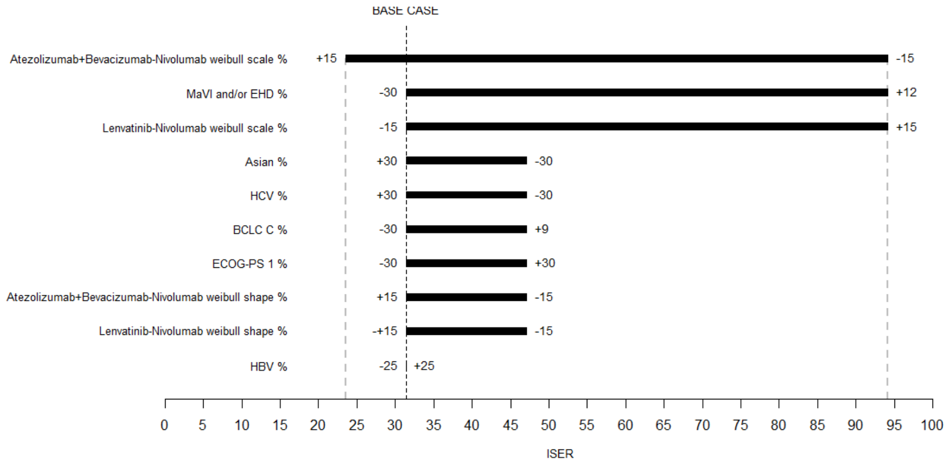

Figure S4. Tornado diagram for a one-way sensitivity analysis of variables which influenced the incremental safety-effectiveness ratio (ISER) of Lenvatinib followed by Nivolumab compared to Atezolizumab + Bevacizuab followed by Nivolumab in patients with advanced hepatocellular carcinoma.

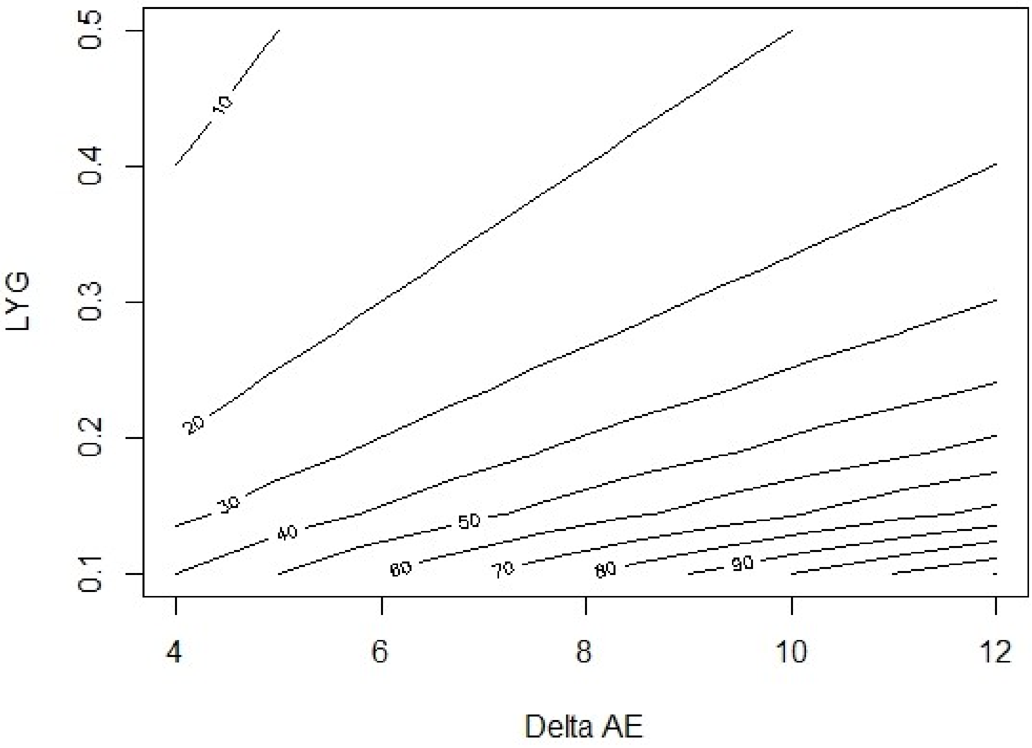

Figure S5. Contour plot of ISER for varying delta SAE and LYG values. Each level curve shows, for a fixed ISER willingness-to-risk threshold (similar to the willingness-to-pay one), how much of the ISER variability area (the one above the corresponding level curve) is favorable to Lenvatinib followed by Nivolumab. Meanwhile, the area below the curve is favorable to Atezolizumab+Bevacizumab followed by Nivolumab.

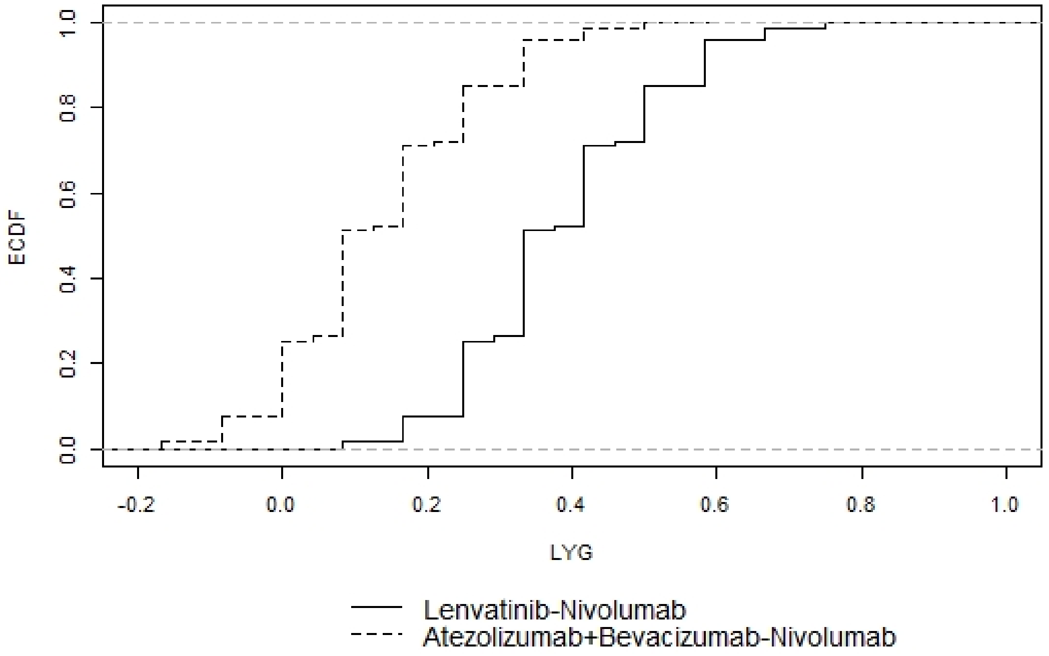

Figure S6. Empirical cumulative density function curves representing the probability that Lenvatinib followed by Nivolumab or Atezolizumab plus Bevacizuab followed by Nivolumab are more effective than Sorafenib followed by Nivolumab in patients with advanced hepatocellular carcinoma.

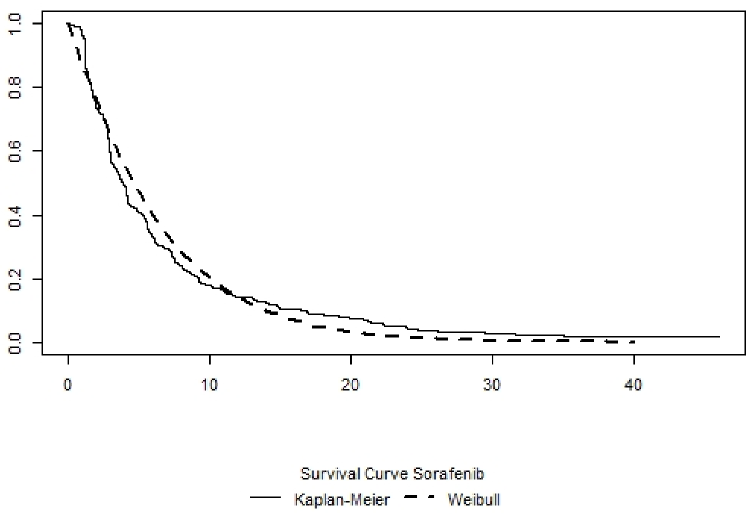

Figure S7. Kaplan-Meier curve with Weibull estimated curve for Sorafenib treatment arms superimposed.

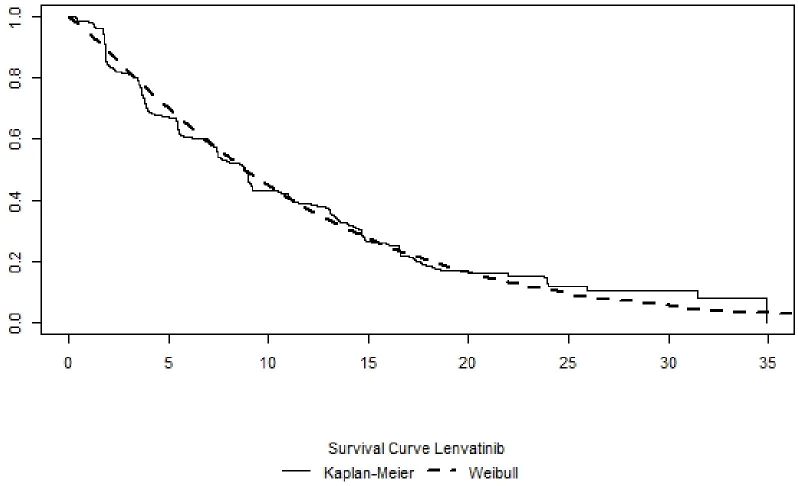

Figure S8. Kaplan-Meier curve with Weibull estimated curve for Lenvatinib superimposed.

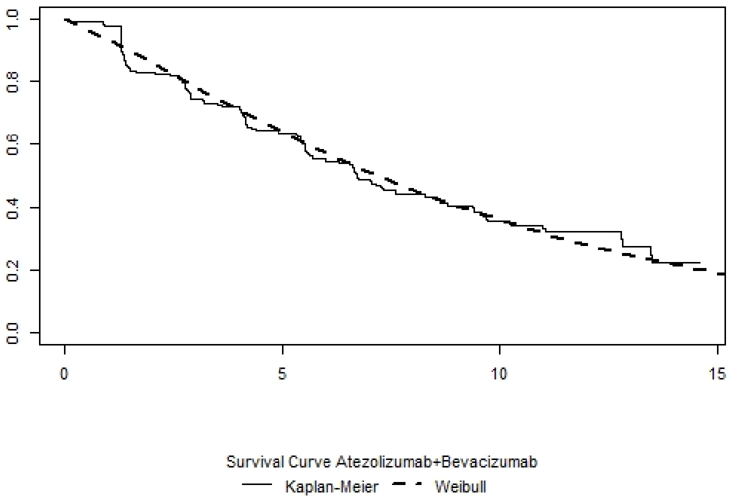

Figure S9. Kaplan-Meier curve with Weibull estimated curve for Atezolizumab + Bevacizumab superimposed.

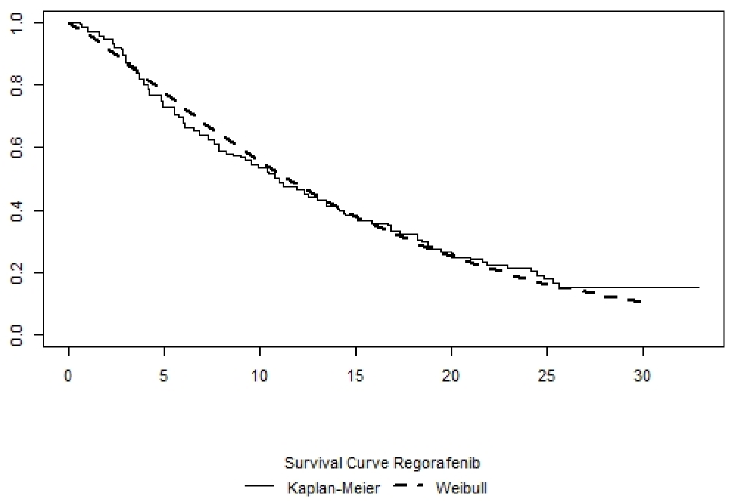

Figure S10. Kaplan-Meier curve with Weibull estimated curve for Regorafenib superimposed.

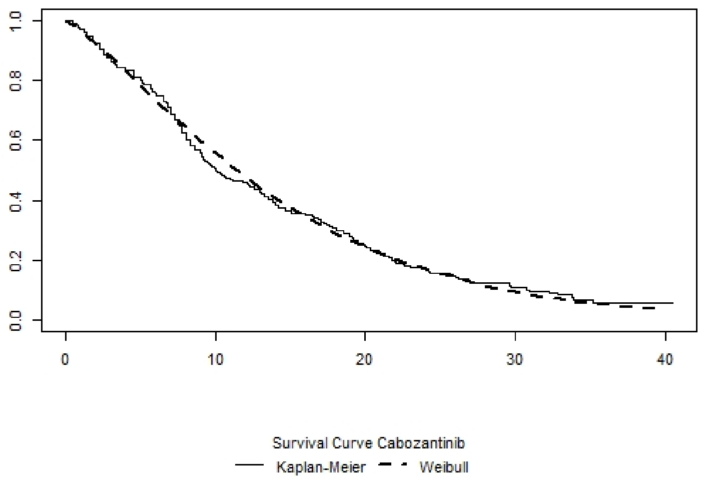

Figure S11. Kaplan-Meier curve with Weibull estimated curve for Cabozantinib superimposed.

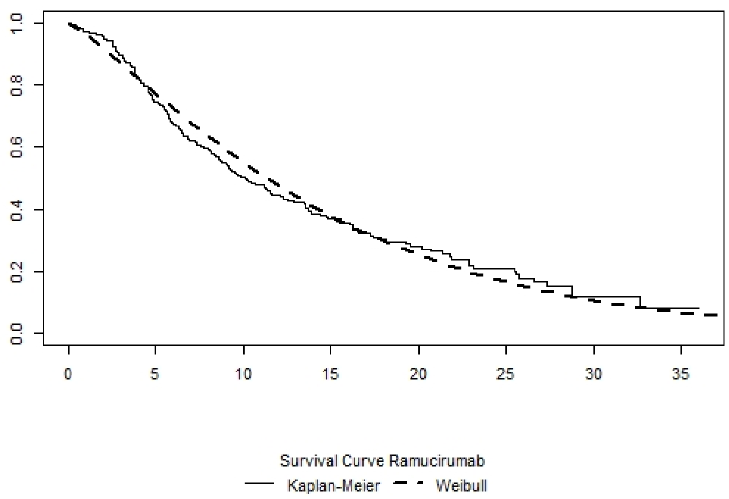

Figure S12. Kaplan-Meier curve with Weibull estimated curve for Ramucirumab superimposed.

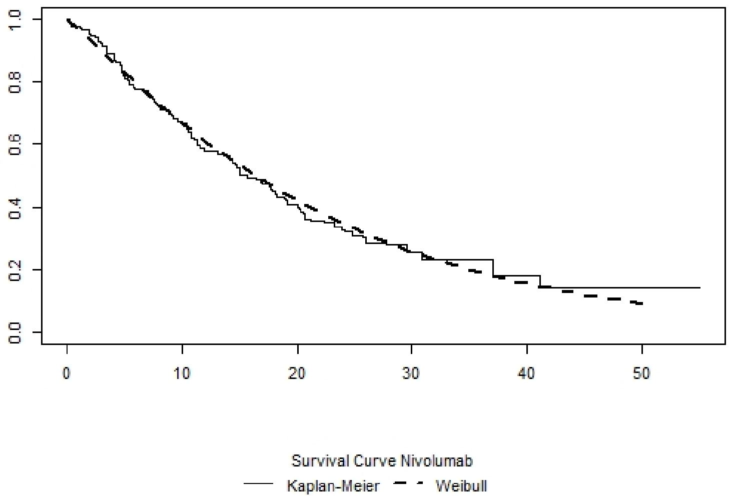

Figure S13. Kaplan-Meier curve with Weibull estimated curve for Nivolumab superimposed.

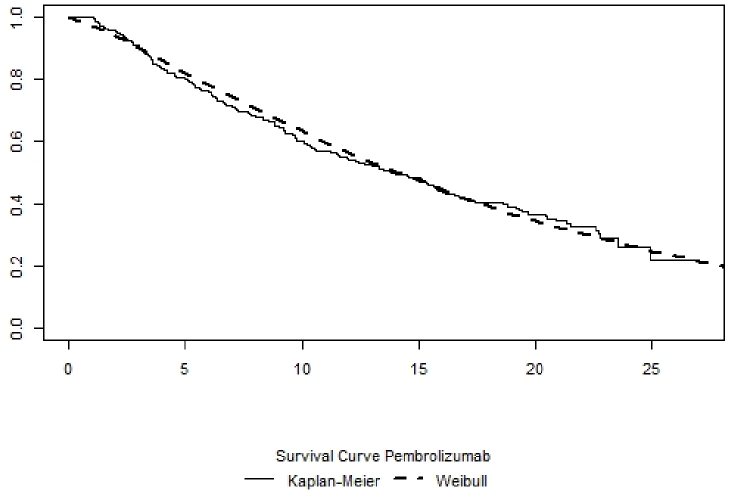

Figure S14. Kaplan-Meier curve with Weibull estimated curve for Pembrolizumab superimposed.

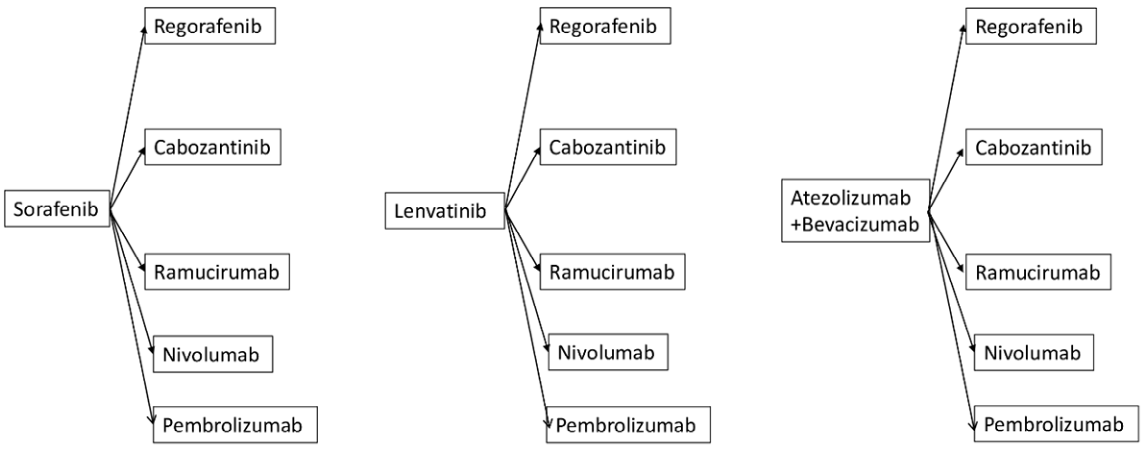

Figure S15. Fifteen sequential systemic treatment strategies deriving from three first-line and five second-line treatments for patients with advanced HCC.

Table S1. Comparison of median overall survival (OS) between Lenvatinib followed by Nivolumab and Atezolizumab + Bevacizumab followed by Nivolumab. The simulated sample size (N) was varied for each treatment arm.

N	Median OS of Lenvatinib-Nivolumab	Median OS of Atezolizumab+Bevacizumab-Nivolumab	*p*-value
100	25	24	0.408
250	25	24	0.239
500	26	25	0.159
750	27	25	0.062
1000	27	25	0.035
1250	27	25	0.016
1500	27	25	0.009
1750	27	25	0.006
2000	27	24	0.002

OS (overall survival) is expressed in months.

© 2020 by the authors. Submitted for possible open access publication under the terms and conditions of the Creative Commons Attribution (CC BY) license (http://creativecommons.org/licenses/by/4.0/).
